# The long non‐coding RNA landscape in triple‐negative breast cancer

**DOI:** 10.1111/cpr.12966

**Published:** 2020-12-13

**Authors:** Wenwen Zhang, Xiaoxiang Guan, Jinhai Tang

**Affiliations:** ^1^ Department of Oncology Nanjing First Hospital, Nanjing Medical University Nanjing China; ^2^ Department of Oncology The First Affiliated Hospital of Nanjing Medical University Nanjing China; ^3^ Department of General Surgery The First Affiliated Hospital of Nanjing Medical University Nanjing China

**Keywords:** biomarkers, LncRNA, LncRNA‐based therapeutics, prognosis, triple‐negative breast cancer

## Abstract

Triple‐negative breast cancer (TNBC) is a type of breast cancer that has a higher risk of distant recurrence and metastasis, leading to a relatively aggressive biological behaviour and poor outcome. So far, the clinical management of TNBC is challenging because of its heterogeneity and paucity of specific targeted therapy. Recently, various studies have identified a lot of differently expressed long non‐coding RNAs (lncRNAs) in TNBC. Those lncRNAs have been reported to play important roles in the multistep process of TNBC tumorigenesis. Here, we review the biological characteristics of lncRNAs, and present the current state of knowledge concerning the expression, function and regulation of lncRNAs in TNBC. Accumulating studies explored the potential lncRNAs‐based therapeutics in TNBC, including the techniques of genetic modification using antisense oligonucleotides, locked nucleic acid and RNA nanotechnology. In current review, we also discuss the future prospects of studies about lncRNAs in TNBC and development of lncRNA‐based strategies for clinical TNBC patients.

## Graphical abstract

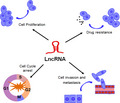

## 1 INTRODUCTION

Breast cancer (BC) is the most frequently diagnosed malignancy and the leading cause of cancer death in females worldwide.^1^ Triple‐negative breast cancer (TNBC) is a subgroup of breast cancers that lack the expression of oestrogen receptor (ER), progesterone receptor (PR) and human epidermal growth factor receptor 2 (HER‐2).^2^ The risk of distant recurrence and metastasis in TNBC patients is substantially higher than in non‐TNBC patients.^3^ The clinical management of TNBC is challenging because of the relatively aggressive biologic behaviour and paucity of specific targeted therapy.^4^ Thus, a better understanding of the regulations and mechanisms of tumorigenesis in TNBC cells and the identification of effective biomarkers for diagnosis and prognosis of TNBC patients are consequently keenly awaited.

LncRNAs, with a length exceeding 200 nucleotides, are non‐protein‐coding transcripts.^5^ Accumulating studies report that lncRNAs expression is dysregulated in various types of cancer including breast cancer, ovarian cancer, hepatocellular carcinoma and many others.^6^, ^7^, ^8^, ^9^, ^10^ Moreover, several lncRNAs have been reported to play crucial roles in various biological processes, including cell proliferation, apoptosis, invasion, differentiation and development.^11^, ^12^, ^13^, ^14^ In TNBC, various studies have identified a lot of dysregulated lncRNAs that play important roles in the process of tumorigenesis through diverse mechanisms. For instance, lncRNAs can act as miRNA ‘sponges’ and compete miRNA‐targeted mRNAs, thereby affecting the miRNA‐mediated gene regulation.^15^, ^16^ This competing endogenous RNAs (ceRNA) mechanisms and network construction, by sequestering miRNAs and sparing their protein‐coding counterparts from post‐translational regulation, have been mainly studied to act as the main molecular mechanism of lncRNA biological function.^15^ Some lncRNAs were reported to assemble with mRNAs to protect them from miRNA action and increase their stability. Some lncRNAs are named scaffold lncRNAs, which could serve as a central platform to assemble with different molecular components such as proteins and RNAs and promote their intermolecular interactions. Moreover, signal lncRNAs have also been reported to interact with transcription factors (TFs) or histone‐modifying enzymes to *cis*‐regulate or *trans*‐regulate the expression of their target genes.^8^ Thus, lncRNAs promise potential diagnostic and prognostic biomarkers, therapeutic targets and improve the clinical benefits for TNBC patients.

Accumulating studies have explored the potential lncRNAs‐based therapeutics in TNBC, including the techniques of genetic modification using antisense oligonucleotides (ASOs), locked nucleic acid (LNA) and RNA nanotechnology. Such as, Jin et al designed eight ASOs targeting LncRNA TROJAN and transfected TNBC cells with ASOs without using any transfection reagents to simulate in vivo conditions. They observed that lung metastasis nodules were significantly reduced in anti‐TROJAN ASO‐treated group than the control group, and the ASO toxicity was limited after detecting the murine blood biochemical indexes.^17^ Hu et al reported that treatment with LINK‐A LNAs could repress cell proliferation in TNBC cells and increase the sensitivity of mammary gland tumours to immunotherapy.^18^ In current review, we accumulated literature to the understanding of lncRNAs biogenesis and function, as well as the latest findings of novel lncRNAs‐based therapeutics in TNBC. We also present the current state of knowledge concerning the expression and regulation of lncRNAs in TNBC, and discuss the future development of lncRNA‐based strategies for clinical TNBC patients.

## 2 BIOLOGICAL CHARACTERISTICS OF LNCRNAS

**FIGURE 1:**
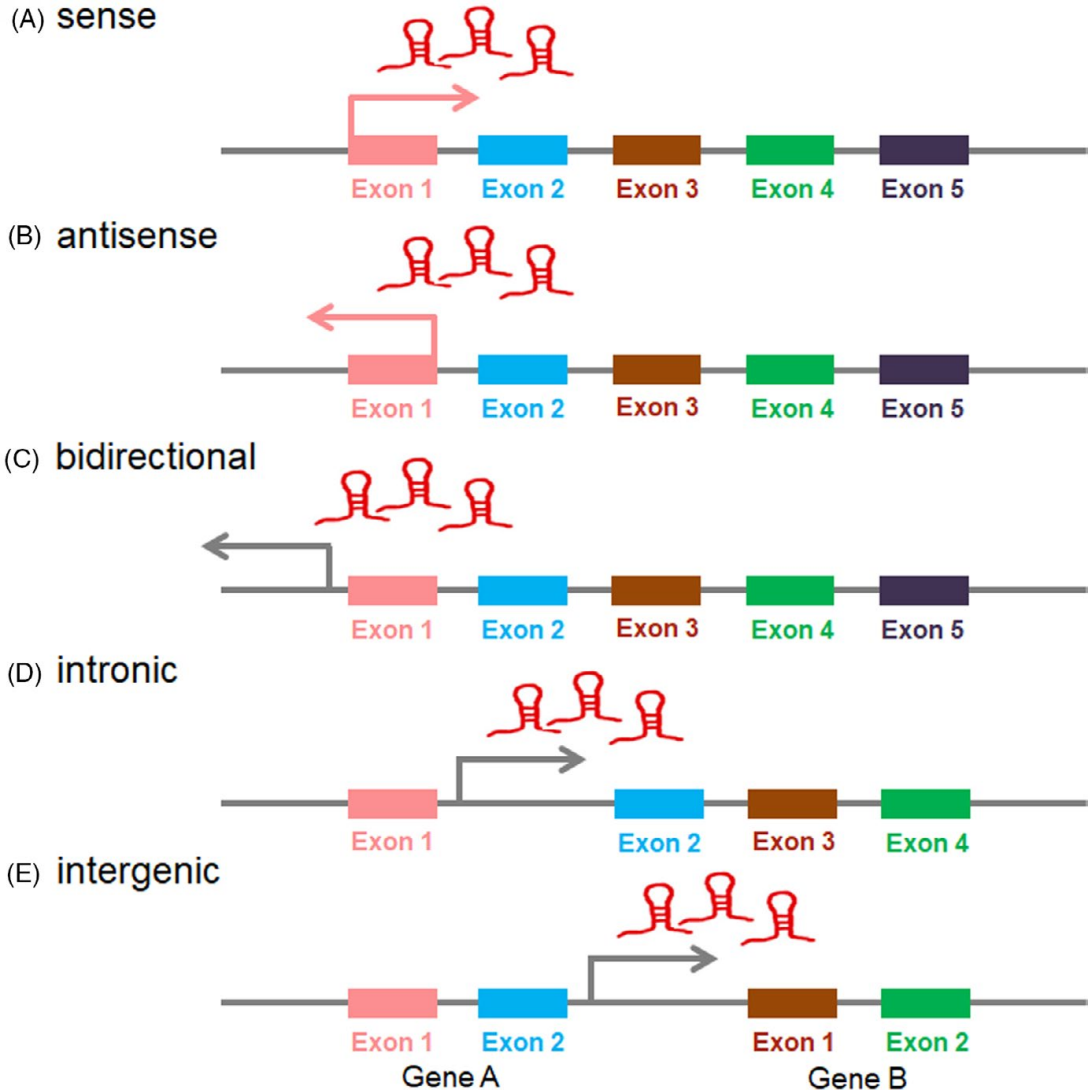
Classification of lncRNAs based on their genomic proximity to neighbouring transcripts

LncRNAs are functionally defined as transcripts >200 nt in length with no protein‐coding potential, many of which are uniquely expressed in differentiated tissues or specific cancer types.^19^ Distinguishing lncRNAs from other protein‐coding mRNAs is not a trivial process. H19, the first lncRNA reported by Brannan et al in 1990, was just defined as not a classical mRNA, and the product of H19 gene was described to be an RNA molecule.^20^ In fact, lncRNAs were first described during the large‐scale sequencing of full‐length cDNA libraries in the mouse.^21^ The number of lncRNAs was reported to outnumber protein‐coding genes, and their sequences cover a larger fraction of the human genome.^22^ LncRNAs may be located within nuclear or cytosolic fractions, and are overlapping with, or interspersed between, multiple coding and non‐coding transcripts.^23^, ^24^ Based on their genomic proximity to neighbouring transcripts, they are classified five categories (Figure 1): (a) sense, overlapping one or more exons of a protein‐coding gene on the same strand; (b) antisense, overlapping one or more exons of a protein‐coding gene on the opposite strand; (c) bidirectional, initiating its expression in close genomic proximity at <1000 base pairs away to a neighbouring coding transcript on the opposite strand; (d) intronic, deriving from an intron of a second transcript; or (e) intergenic, acting as an independent unit within the genomic interval between two genes.^25^, ^26^ LncRNAs were initially thought to be the products of an inconsequential transcription resulting from low RNA polymerase fidelity.^27^ It is now widely recognized that lncRNAs could identify cellular pathologies such as cancer, provide prognostic value, or even inform therapeutic options for cancer patients, by serving as signals of specific cellular states or readouts of active cellular programmes.^28^ Recent studies have shown that lncRNAs can regulate gene expression at different levels, including chromatin modification, transcription and post‐transcriptional regulation.^29^ LncRNAs were reported to regulate several biological processes such as cell proliferation, apoptosis, cell cycle, cell invasion and metastasis, cellular differentiation, chromatin modification and nuclear‐cytoplasmic trafficking.^30^ It has been suggested that the involvement of lncRNAs in human diseases could be far more prevalent than previously known.^31^ Recently, lncRNAs‐related studies in cancer increased dramatically and have become one of the hottest topics in RNA biology.

## 3 PROFILES OF LNCRNAS EXPRESSION IN TNBC

Recently, abnormal expression of many lncRNAs has been found in almost all tumours in humans, including TNBC. However, our understanding of lncRNAs remains significantly less mature than mRNAs, or even miRNAs. Next generation sequencing (NGS) is a DNA sequencing technology, which could perform sequencing of millions of small fragments of DNA in parallel. These fragments are then pieced together by mapping the individual reads to the human reference genome.^32^ NGS is now used to sequence entire genomes or constrained to specific areas of interest to get the population genomic and gene expression differences in a large array of organisms.^32^ Thus, NGS technologies may help researchers to accelerate the identification and characterization of important, yet‐to‐be‐annotated functional lncRNAs in TNBC.

In recent years, researchers got a lot of abnormally expressed lncRNAs in TNBC patients or cells using public databases based on the NGS technologies. Tian et al found a total of 1034 dysregulated lncRNAs in the two TNBC microarrays from the Gene Expression Omnibus (GEO) database. Among them, 537 lncRNAs were significantly correlated with 451 protein‐coding genes, which were mainly enriched in terms including cell division, cell cycle, and involved in PI3K‐Akt, MAPK, ErbB family and p53 signalling pathways.^33^ In addition, further literatures related to the lncRNA expressions profiles also detected a series of dysregulated lncRNAs in TNBC.^34^, ^35^, ^36^, ^37^, ^38^, ^39^, ^40^

Previous studies have shown that lncRNAs can act as miRNA ‘sponges’ and compete miRNA‐targeted mRNAs, thereby affecting the miRNA‐mediated gene regulation.^15^, ^16^ This crosstalk forms a complex post‐transcriptional regulatory network including mRNAs, lncRNAs, called competing endogenous RNAs (ceRNA) network.^15^, ^16^ ceRNA‐mediated regulatory mechanisms are reported to be an important pathway for lncRNAs‐modulated post‐transcriptional regulation in TNBC. Such as, Le et al developed a complex ceRNA network in TNBC using microarray mRNA and lncRNA expression data obtained from The Cancer Genome Atlas (TCGA) database and two GEO databases.^41^ As a result, they identified differentially expressed 4565 mRNAs, 427 miRNAs and 4852 lncRNAs, and constructed ceRNA network using 37 lncRNAs, 28 miRNAs and 16 mRNAs. On the basis of establishing the ceRNA network, they found that two mRNAs expression are correlated with prognosis of TNBC patients.^41^ Similarly, Liu et al also constructed a ceRNA network based on analysis of differentially expressed RNAs between 150 TNBC tissues and 823 non‐TNBC tissues downloaded from TCGA database.^42^ They identified 190 differentially expressed lncRNAs, 48 differentially expressed mRNAs and 13 differentially expressed miRNAs in this ceRNA network. They concluded that eight lncRNAs and one mRNA could act as prognostic factors in TNBC, using survival analysis and receiver operating characteristic (ROC) curve creation in the network.^42^ Additionally, they found that lncRNA OSTN‐AS1 was primarily related to immunologic function, including immune cell infiltration and immune‐related markers co‐expression.^42^ Song et al also constructed a ceRNA network of TNBC using TCGA database and revealed 686 mRNAs, 26 miRNAs and 50 lncRNAs as key molecules for high risk of TNBC.^43^ At the same time, the ceRNA crosstalk network of TNBC constructed by Yuan et al contains 22 hub mRNAs, and 14 key differentially expressed lncRNAs.^44^ Jiang et al developed an integrated ceRNA network signature based on three mRNA (FCGR1A, RSAD2, CHRDL1) and two lncRNA (HIF1A‐AS2 and AK124454), using transcriptome microarrays for 33 paired TNBC and adjacent normal breast tissue.^45^ They also found that the prognostic and predictive accuracy of this ceRNA signature was better than clinicopathological parameters to predict tumour recurrence and the benefit of taxane chemotherapy in TNBC.^45^Taken together, the ceRNA co‐regulatory network could help us understand the potential characteristics of biological function and pathological roles of lncRNAs in the development and progression of TNBC.

## 4 ROLES OF LNCRNAS IN TNBC

**FIGURE 2:**
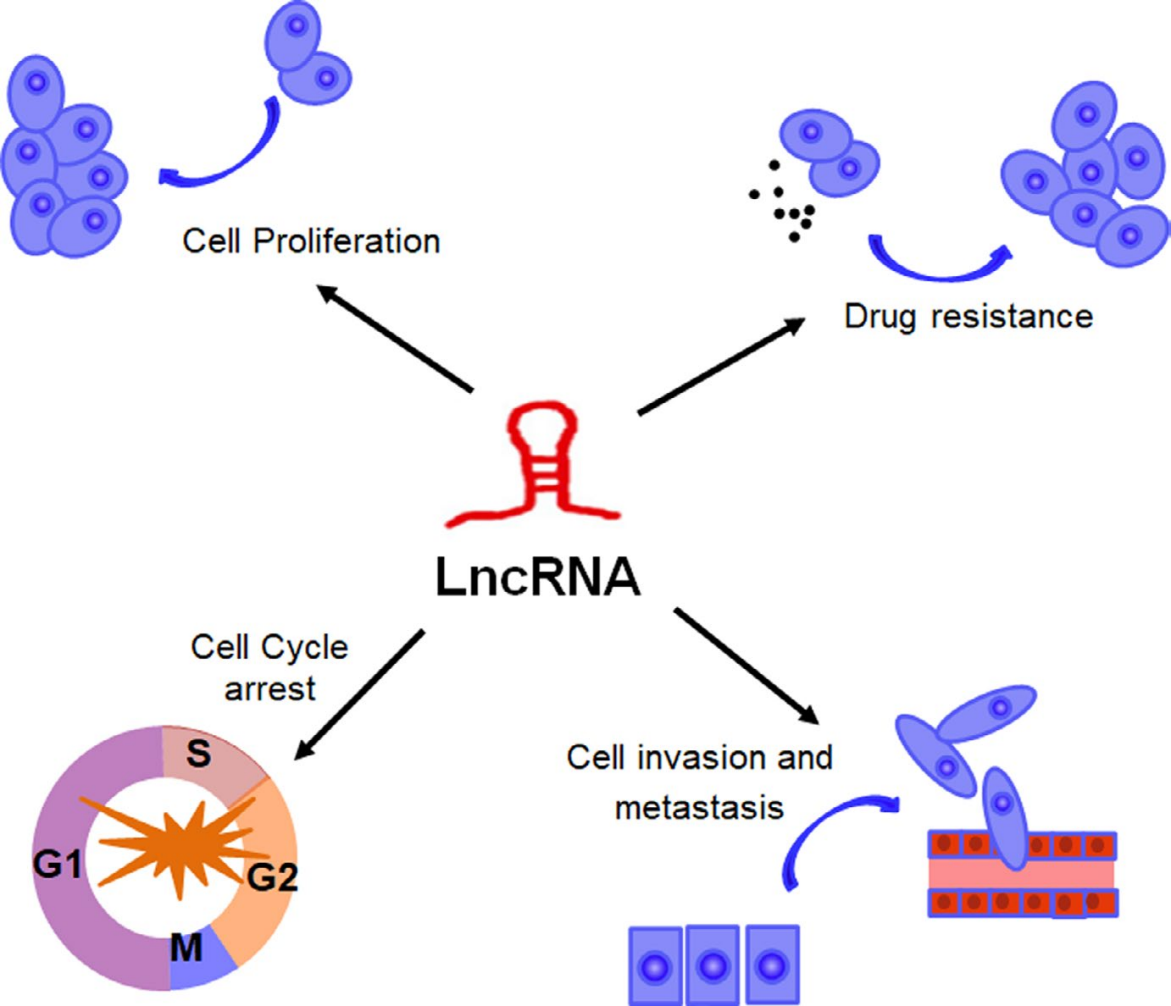
The biological function of lncRNAs in TNBC

To date, numerous lncRNAs have been identified to dysregulated express and play an important role in the biological function of TNBC, including cellular proliferation, apoptosis, cell cycle, migration, invasion, angiogenesis and drug resistance (Table 1). In this chapter, we will provide an overview of lncRNA biological function in TNBC (Figure 2).

**TABLE 1:** Identified lncRNAs in TNBC

lncRNAs	Expression	Biological function	Potential Targets	References
GAS5	Down	Inhibit cell proliferation and invasion; Promote cell apoptosis; Inhibit paclitaxel, cisplatin, adriamycin and PI3K/mTOR inhibitor resistance	miR‐378a‐5p/SUFU, miR‐196a‐5p	47, 48, 49, 108
LINC02095	Up	Promote cell proliferation	SOX9	^55^
HOTAIR	Up	Promote cell migration and invasion; imatinib and lapatinib resistance	miR‐148a; LEF1/TCF4	92, 93, 109
WT1‐AS	Down	Inhibit cell migration and invasion	TGF‐β1	^96^
LINC00096	Up	Promote cell proliferation and invasion	miR‐383‐5p/RBM3	^50^
DRHC	Down	Inhibit cell proliferation	HOTAIR	^60^
HEIH	Up	Promote cell proliferation Inhibit cell apoptosis	miR‐4458/SOCS1	^51^
LUCAT1	Up	Promote cell proliferation, cell cycle progression and metastasis; Inhibit cell apoptosis	miR‐5702	^64^
CCAT1	Up	Promote cell proliferation, migration, and invasion	miR‐218/ZFX	^77^
ASRPS	Down	Inhibit angiogenesis	STAT3	^125^
HAND2‐AS1	Down	Inhibit cell proliferation	RUNX2	^65^
LINC01133	Up	Promote cell stem cell (CSC)‐like phenotypic traits	KLF4	^119^
LINC01096	Up	Promote cell proliferation, migration, and invasion; Inhibit cell apoptosis	miR‐3130‐3p	^97^
PAPAS	Up	Promote cell migration and invasion	miR‐34a	^83^
HCP5	Up	Promote cell proliferation; Inhibit cell apoptosis	miR‐219a‐5p/BIRC3	^52^
NRAD1	Up	Promote cell proliferation and CSC‐like phenotypic traits	‐	^122^
LINK‐A	Up	Promote immunotherapy resistance; AKT inhibitors resistance; glycolysis reprogramming	PI3K/GPCR; Akt; HIF1α	18, 110, 127
MIR503HG	Down	Inhibit cell migration and invasion	miR‐103/OLFM4	^78^
AWPPH	Up	Promote cell proliferation; Promote carboplatin resistance	miR‐21; FZD7	63, 112
PTCSC3	Down	Inhibit cell proliferation	H19	^61^
NRON	Down	Inhibit cell proliferation	snaR	^62^
sONE	Down	Inhibit cell proliferation, migration, and invasion	miR‐34a/15a/16, let‐7a, TP53/c‐Myc; NOS3	84, 85
NAMPT‐AS	Up	Promote cell metastasis	miR‐548b‐3p/ NAMPT	^79^
DANCR	Up	Promote cell proliferation and invasion, and CSC‐like phenotypic traits	miR‐216a‐5p; RXRA; EZH2, CD44, ABCG2; Nanog, SOX2, and OCT4	56, 98, 120
NEAT1	Up	Inhibit cell apoptosis; Promote cell cycle progression; Promote cisplatin and paclitaxel resistance and cancer stemness	‐	^75^
TROJAN	Up	Promote cell proliferation and invasion	ZMYND8	^17^
POU3F3	Up	Promote cell proliferation; Inhibit cell apoptosis	Caspase‐9	^66^
NEF	Down	Inhibit cell migration and invasion	miR‐155	^99^
ZEB2‐AS1	Up	Promote cell proliferation, metastasis and EMT	ZEB2	^88^
LncKLHDC7B	Down	Inhibit cell migration and invasion; Promote cell apoptosis	KLHDC7B	^100^
HIF1A‐AS2	Up	Promote cell migration and invasion	‐	^101^
LINC00339	Up	Promote cell proliferation; Inhibit cell cycle arrest, apoptosis	miR‐377‐3p/HOXC6	^53^
LINC00152	Up	Promote cell proliferation and invasion; Inhibit cell apoptosis	PTEN, BRCA1	57, 58
AFAP1‐AS1	Up	Promote EMT	Wnt/β‐catenin	^81^
PDCD4‐AS1	Down	Inhibit cell proliferation and migration	PDCD4	^59^
HOST2	Down	Inhibit cell proliferation	let‐7b/CDK6	^54^
BORG	Up	Promote doxorubicin resistance	RPA1	^115^
PVT1	Up	Promote cell proliferation and migration, EMT	p21, KLF5/β‐catenin	89, 90
H19	Up	Promote paclitaxel resistance and CSC‐like phenotypic traits	Akt	114, 123
TP73‐AS1	Up	Promote cell vasculogenic mimicry	miR‐490‐3p/TWIST1	^126^
TUG1	Down	Enhance cisplatin sensitivity	miR‐197/NLK	^113^
MIR100HG	Up	Promote cell proliferation, Inhibit cell cycle arrest	p27	^72^
LINC01638	Up	Promote cell proliferation, metastasis and CSC‐like phenotypic traits	c‐Myc	^121^
ARNILA	Down	Promote EMT, invasion and metastasis	miR‐204/SOX4	^91^
LINC‐ZNF469‐3	Up	Promote cell invasion, stemness properties and lung metastasis	miR‐574‐5p/ZEB1	^87^
ROR	Up	Promote cell invasion and metastasis	miR‐145/MUC1; miR‐145/ARF6	102, 103
AIRN	Down	Inhibit cell migration and invasion	Wnt/β‐catenin/mTOR/PI3K	^104^
MANCR	Up	Promote cell proliferation, Inhibit DNA damage	‐	^128^
RMST	Down	Inhibit cell proliferation, invasion and migration; Promote cell apoptosis, and regulate cell cycle.	‐	^76^
SKAI1BC	Up	Promote cell migration and invasion	KAI1	^105^
ANRIL	Up	Promote cell proliferation, Inhibit cell apoptosis	miR‐199a	^67^
MALAT1	Up	Promote cell proliferation, cell cycle arrest, and invasion; Inhibit cell apoptosis	miR‐129‐5p; miR‐1/Slug; miR‐448/KDM5B	68, 106, 149
SNHG12	Up	Promote cell proliferation and migration, Inhibit cell apoptosis	MMP13	^94^
HULC	Up	Promote cell metastasis	MMP‐2, MMP‐9	^107^
SNAR	Up	Promote cell proliferation, migration and invasion	‐	^95^
LINP1	Up	Promote DNA DSB repair, and radiotherapy resistance	Ku80	^117^
PCAT6	Up	radiotherapy resistance	miR‐185‐5p/TPD52	^118^

### 4.1 lncRNAs involved in the regulation of cell proliferation and apoptosis

Cancer has been considered to be the result of accumulated gene mutations, which led to uncontrolled cell proliferations. And deregulated cell proliferation and inhibition of cell apoptosis lie at the heart of tumour development.^46^ The role of lncRNAs in the regulation of TNBC cell proliferation and apoptosis has also been widely investigated. ceRNA mechanisms and network construction, by sequestering miRNAs and sparing their protein‐coding counterparts from post‐translational regulation, have been mainly studied to act as the main molecular mechanism of lncRNA biological function.^15^ For example, lncRNA GAS5 was reported to promote apoptosis and inhibit proliferation of TNBC cells by targeting miR‐196a‐5p and miR‐378a‐5p/SUFU signalling.^47^, ^48^, ^49^ LINC00096 promoted cell proliferation by sponging miR‐383‐5p and regulating RBM3 expression in TNBC.^50^ LncRNA HEIH was shown to regulate cell proliferation and apoptosis through miR‐4458/SOCS1 axis in TNBC.^51^ LncRNA HCP5 could also promote cell proliferation and inhibit cell apoptosis as a ceRNA to regulate BIRC3 by sponging miR‐219a‐5p.^52^ LINC00339 promoted cell proliferation and inhibited cell apoptosis through miR‐377‐3p/HOXC6 signalling pathway.^53^ Knockdown of lncRNA HOST2 could inhibit the proliferation of TNBC cells via regulation of the let‐7b/CDK6 axis.^54^

There are also other molecular mechanisms of LncRNAs. Some lncRNAs assemble with mRNAs to protect them from miRNA action and increase their stability. Some lncRNAs are named scaffold lncRNAs, which could serve as a central platform to assemble with different molecular components such as proteins and RNAs and promote their intermolecular interactions. Signal lncRNAs have also been reported to interact with transcription factors (TFs) or histone‐modifying enzymes to *cis*‐regulate or *trans*‐regulate the expression of their target genes.^8^ For instance, in TNBC, Tariq et al revealed that LINC02095 promotes breast cancer proliferation by facilitating the expression of oncogenic transcription factor, SOX9.^55^ LncRNA DANCR was reported to bind with RXRA and increase its serine 49/78 phosphorylation, leading to activating PI3K/Akt signalling and TNBC cell proliferation.^56^ Shen et al demonstrated that LINC00152 obviously enhanced NEDD4‐1‐mediated ubiquitination and degradation of PTEN protein in TNBC.^57^ Meanwhile, Wu et al also revealed that LINC00152 could enhance TNBC tumorigenesis by inactivation of the BRCA1/PTEN through DNA methyltransferases.^58^ Besides, LncRNA PDCD4‐AS1 was reported to stabilize PDCD4 RNA by forming RNA duplex and regulate the interaction between PDCD4 RNA and RNA decay promoting factors such as HuR.^59^

Several studies have indicated that LncRNAs could also play an important role in the TNBC cell proliferation and apoptosis process by regulating other LncRNAs. LncRNA DRHC was shown to inhibit TNBC cells proliferation by down‐regulating the expression of lncRNA HOTAIR, while HOTAIR did not affect the expression level of DRHC.^60^ Similarly, LncRNA PTCSC3 overexpression led to down‐regulated lncRNA H19 in TNBC cells, while H19 overexpression did not affect PTCSC3 expression.^61^ LncRNA NRON overexpression inhibited cancer cell proliferation and down‐regulated lncRNA snaR in TNBC, while snaR overexpression did not significantly affect NRON expression.^62^ There are other lncRNAs involved in the process of TNBC cell proliferation and apoptosis, including AWPPH, LUCAT1, HAND2‐AS1, POU3F3, MALAT1, ANRIL.^63^, ^64^, ^65^, ^66^, ^67^, ^68^ These lncRNAs could be potential targets for further mechanistic studies to establish their functional role in TNBC cell proliferation and apoptosis.

**FIGURE 3:**
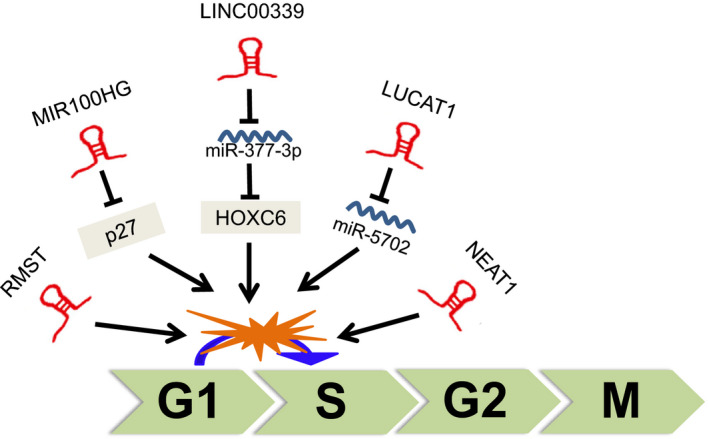
lncRNAs involved in the regulation of TNBC cell cycle

Cell cycle progression is regulated by cyclin‐dependent kinases (CDKs), which are activated by cyclin binding and inhibited by CDK inhibitors.^69^ p27, an inhibitor of CDK, binds not only to the cyclin E/CDK2 complex, but also to the cyclin D/CDK4,6 complexes, involving in the regulation of the cell cycle.^70^, ^71^ LncRNA MIR100HG was reported to inhibit cell arrest in the G_1_ phase, through binding to p27 to form RNA‐DNA triplex structures at 275‐352 nt, 462‐557 nt and 2635‐2688 nt.^72^ It was showed that lncRNA LUCAT1 plays a key role in cell cycle G_1_ arrest by regulating the expression of cyclin D1, CDK4 in clear cell renal cell carcinoma (ccRCC) ^73^ and the expression of p21, p57 in non‐small‐cell lung cancer (NSCLC).^74^ In TNBC, LUCAT1 was also shown to contribute to accelerate cell cycle progression through modulating miR‐5702.^64^ Besides, Wang et al reported that LINC00339 inhibited cell cycle arrest at G_0_/G_1_ phase by sponging to miR‐377‐3p and activating miR‐377‐3p/HOXC6 signalling pathway in TNBC.^53^ Shin et al revealed that LncRNA NEAT1 conferred oncogenic role by regulating cell cycle progression in TNBC cells.^75^ LncRNA RMST was also shown to induce the block of G_0_/G_1_ phase in TNBC.^76^ Taken together, researches about lncRNAs in the regulation of cell cycle in TNBC are preliminary (Figure 3). Maybe, lncRNAs profiles to identify the abnormally expressed lncRNAs and further mechanistic studies to investigate the role of lncRNAs in the regulation of cell cycle progression in TNBC are needed.

### 4.2 lncRNAs involved in the regulation of cell invasion and metastasis

Metastasis is the major cause of cancer‐related deaths. It has been increasingly recognized that lncRNAs play important roles in tumour invasiveness and metastasis. Overexpression of GAS5 was shown to undermine the tumour promotion effect induced by ectopic expression of miR‐196a‐5p, including cell invasion and FOXO1/PI3K/Akt signal pathway activation.^49^ TROJAN can bind to metastasis‐repressing factor, ZMYND8 and increase its degradation through the ubiquitin‐proteasome pathway.^17^ Han et al reported that lncRNA CCAT1 could promote TNBC cells migration and invasion by suppressing miR‐218/ZFX signalling.^77^ In addition, lncRNA MIR503HG was reported to inhibit cell migration and invasion via miR‐103/OLFM4 axis in TNBC.^78^ LncRNA NAMPT‐AS promoted TNBC cell metastasis and regulated autophagy, through epigenetically regulating NAMPT expression. NAMPT‐AS could recruit POU2F2 to activate the transcription of NAMPT, or serve as a ceRNA to rescue NAMPT degradation from miR‐548b‐3p in TNBC.^79^

Epithelial‐mesenchymal transition (EMT) has been involved in carcinogenesis and confers metastatic properties upon cancer cells by enhancing cell mobility, invasion and resistance to apoptotic stimuli.^80^ Zhang et al revealed that AFAP1‐AS1 could activate Wnt/β‐catenin pathway to promote tumorigenesis and cell invasion by inducing the expression of c‐myc and EMT‐related molecules in TNBC.^81^ MiR‐34a was reported to implicate in certain EMT‐associated signal pathways to repress tumorigenesis, cancer progression and metastasis.^82^ LncRNA PAPAS was shown to promote migration and invasion of TNBC cells by down‐regulating miR‐34a.^83^ LncRNA sONE was also reported to repress endothelial nitric oxide synthase (eNOS)‐induced nitric oxide (NO) production, regulating TP53 and c‐Myc proteins levels and finally altering the levels of a panel of tumour‐suppressor miRNAs, including miR‐34a, miR‐15, miR‐16 and let‐7a.^84^ Besides, sONE was also shown to inhibit H_2_S‐induced TNBC cell migration and invasion through activating sONE/NOS3/NO signalling axis.^85^ ZEB1 and ZEB2 belong to the ZEB family transcription factors, which play a pivotal role in the process of EMT.^86^ LncRNA LINC‐ZNF469‐3 was reported to enhance invasion ability and stemness properties, and promote lung metastasis through miR‐574‐5p/ZEB1 axis in TNBC.^87^ LncRNA ZEB2‐AS1 was shown to promote TNBC cell metastasis by positively regulating ZEB2 expression and activating the EMT via the PI3K/Akt/GSK3β/ZEB2 signalling pathway.^88^ Another lncRNA, PVT1, was reported to promote EMT and cell migration via regulating p21 and KLF5/β‐catenin signalling in TNBC.^89^, ^90^

**FIGURE 4:**
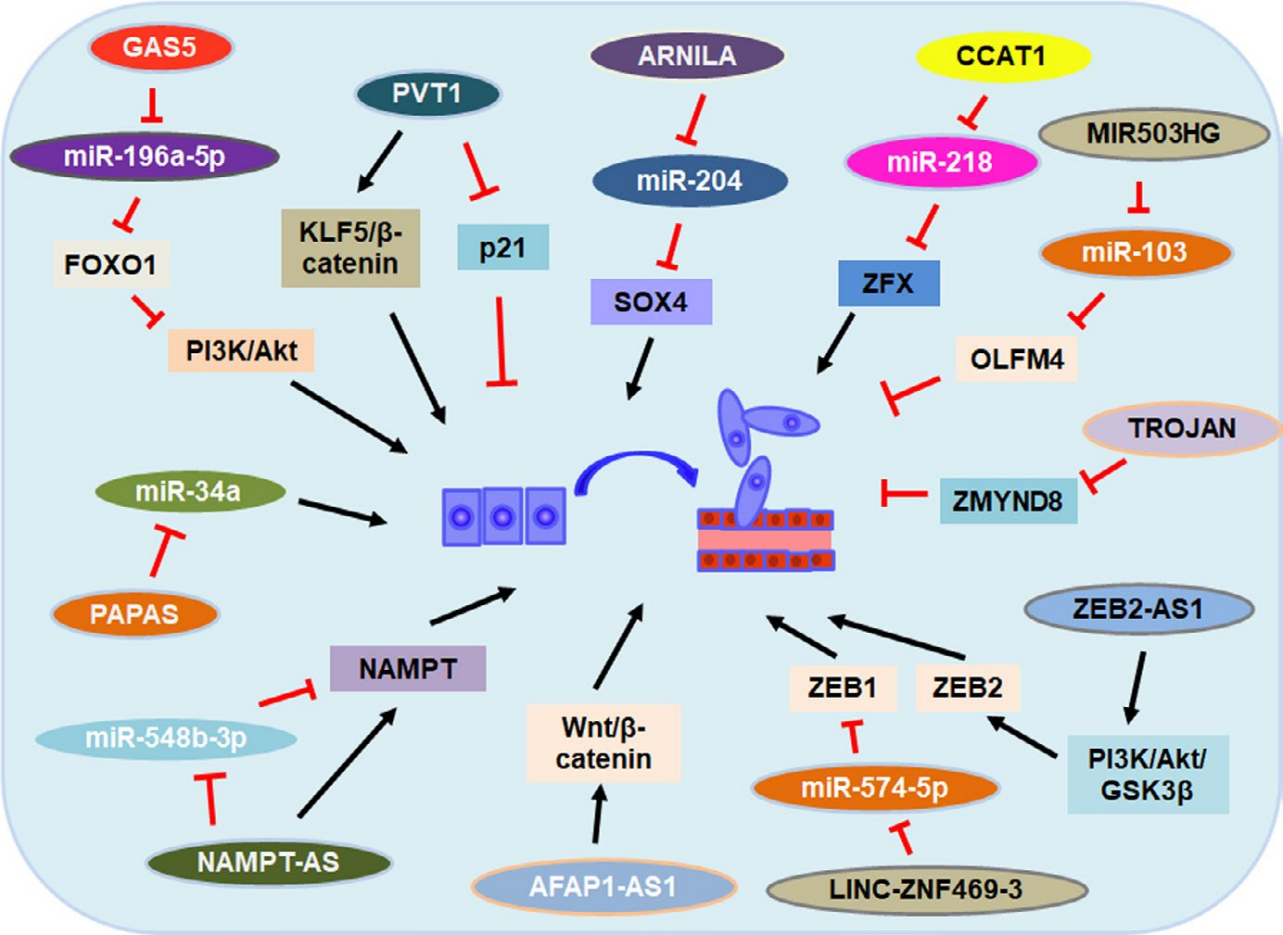
lncRNAs involved in the regulation of TNBC cell invasion and metastasis

The results of our studies also showed that lncRNA androgen receptor (AR) negatively induced long non‐coding RNA (ARNILA) could promote EMT, invasion and metastasis of TNBC, by functioning as a ceRNA for miR‐204 to facilitate expression of its target gene Sox4.^91^ There are other lncRNAs involved in the process of TNBC invasion and metastasis, including HOTAIR, SNHG12, SNAR, WT1‐AS, LINC01096, DANCR, NEF, HIF1A‐AS2, LncKLHDC7B, ROR, AIRN, RMST, MALAT1, SKAI1BC, HULC.^76^, ^107^ Taken together, these studies revealed that lncRNAs play an important role in the regulation of cell invasion and metastasis in TNBC (Figure 4).

### 4.3 lncRNAs involved in the regulation of drug resistance

**FIGURE 5:**
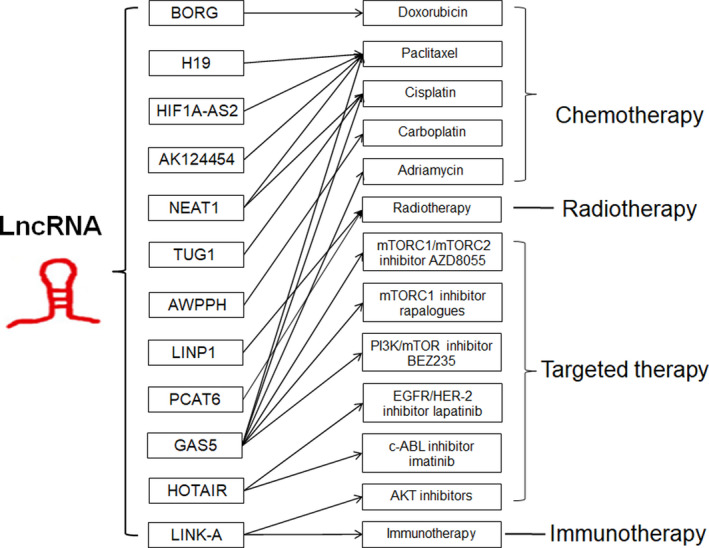
lncRNAs involved in the regulation of drug resistance in TNBC

Emerging evidences suggest that lncRNAs could implicate in regulation of drug resistance by targeting different genes in TNBC (Figure 5). LncRNA growth‐stasis‐specific transcript 5 (GAS5) is the most widely studied lncRNA involved in the regulation of various drug resistance. The expression level of GAS5 in TNBC patients was reported to associate with tumour resistance to several chemotherapeutic drugs, including adriamycin, paclitaxel and cisplatin.^47^, ^48^ In addition, GAS5 expression could reduce the sensitivity to not only mTORC1 inhibitor rapalogues, but also dual mTORC1/mTORC2 inhibitor AZD8055.^108^ Nevertheless, they displayed a significant increase in response to the dual PI3K/mTOR inhibitor, BEZ235.^108^ Considering the important role of GAS5 in the sensitivity of multiple drugs, GAS5 may be a potential biomarker for monitoring prognosis of TNBC patients.

Besides, HOTAIR expression was shown to be transcriptionally repressed by the combined treatment of EGFR/HER‐2 inhibitor lapatinib plus c‐ABL inhibitor imatinib. Enforced expression of HOTAIR conferred increased resistance to the dual treatment by recruitment of β‐catenin to the HOTAIR promoter at the LEF1/TCF4‐binding site.^109^ Another lncRNA, LINK‐A (long intergenic non‐coding RNA for kinase activation, also called LINC01139), could directly interact with the AKT pleckstrin homology (PH) domain and PIP_3_, facilitating AKT‐PIP_3_ interaction and consequent enzymatic activation.^110^ LINK‐A‐dependent AKT hyperactivation led to resistance to AKT inhibitors, while genomic deletions of the LINK‐A PIP_3_‐binding motif dramatically sensitized TNBC cells to AKT inhibitors.^110^ Immunotherapy, including programmed cell death protein‐1 and programmed death ligand‐1 (PD‐1/PD‐L1) blockade, has been demonstrated to inhibit cancer progression and validated with the clinical success for the treatment of a variety of human cancers.^111^ Hu and colleagues demonstrated that LINK‐A could also regulate the immunosurveillance in TNBC via LINK‐A‐PKA‐TRIM71 signalling axis.^18^ Patients with PD‐1 blockade‐resistant TNBC exhibited elevated LINK‐A levels, and LINK‐A locked nucleic acids treatment sensitized mammary gland tumours to immune checkpoint blockers.^18^

There are other lncRNAs involved in the regulation of drug resistance, including chemotherapy drugs paclitaxel, doxorubicin, cisplatin, carboplatin and radiotherapy resistance. For example, LncRNA AWPPH could improve cancer cell viability under carboplatin treatment, while lncRNA AWPPH small interfering RNA (siRNA) silencing led to increased chemosensitivity.^112^ HIF1A‐AS2 and AK124454 were showed to contribute paclitaxel resistance in TNBC cells.^45^ LncRNA TUG1 was shown to sponge miR‐197, induce expression of NLK and inactivate WNT signalling pathway, thus increasing cisplatin sensitivity of TNBC cells.^113^ LncRNA H19 was also reported to confer paclitaxel resistance, while knockdown of H19 might restore the chemosensitivity in paclitaxel‐resistant TNBC by mediating the AKT signalling pathway.^114^ LncRNA NEAT1 was shown to mediate paclitaxel and cisplatin resistance in TNBC.^75^ Besides, lncRNA BORG led to doxorubicin resistance via binding to RPA1 and activating the NF‐κB signalling axis.^115^ DNA repair is a series of processes by which damaged DNA is identified and corrected in cells. This process is essential to genomic integrity and is involved in tumorigenesis.^116^ LncRNA LINP1 was reported to enhance repair of DNA double‐strand breaks (DSB) by acting as a scaffold linking Ku80 and DNA‐dependent protein kinase catalytic subunit (DNA‐PKcs), thereby coordinating the NHEJ pathway, a key determinant of ionizing radiation (IR) resistance.^117^ Importantly, blocking LINP1 could increase the sensitivity of the tumour‐cell response to radiotherapy in TNBC.^117^ Additionally, knockdown of lncRNA PCAT6 promoted the radiosensitivity of TNBC cells through regulating miR‐185‐5p/TPD52 axis.^118^ Taken together, these studies evoke the potential of altering lncRNAs expression in future to represent a novel therapeutic approach to reverse drug resistance or radiotherapy resistance in TNBC patients. However, further studies and mechanistic investigations of the regulation mechanism of lncRNAs‐mediated drug resistance in TNBC are needed in the future.

### 4.4 Others

Several recent studies also demonstrated that lncRNAs could implicate in other malignant processes, including angiogenesis and cancer stemness. For example, it was reported that mesenchymal stem/stromal cells (MSCs) strongly induced the lncRNA LINC01133 in neighbouring TNBC cells.^119^ LINC01133 promoted phenotypic and growth characteristics of cancer stem cell‐like cells, and that it was a direct mediator of the MSC‐triggered miR‐199a/FOXP2 pathway and pluripotency‐determining gene Kruppel‐Like Factor 4 (KLF4) in TNBC models.^119^ LncRNA DANCR was shown to promote the expression of TNBC cancer stem cell markers through repressing the binding of EZH2 on the promoters of CD44 and ABCG2.^120^ LINC01638 was reported to maintain the mesenchymal traits of TNBC cells, including an enriched EMT signature and cancer stem cell‐like state, through interacting with c‐Myc to prevent SPOP‐mediated c‐Myc degradation, and activate MTDH/Twist1 signalling.^121^ Furthermore, there are other lncRNAs reported to involve in the regulation of TNBC stemness, including NEAT1, LINC‐ZNF469‐3, NRAD1, H19.^75^, ^87^, ^122^, ^123^ TNBC patients demonstrate enhanced angiogenesis when compared with non‐TNBC patients.^124^ Wang et al recently reported that lncRNA ASRPS could directly bind to STAT3 in the coiled coil domain (CCD) and inhibit STAT3 phosphorylation, leading to reduced expression of VEGF and reduced angiogenesis.^125^ Vasculogenic mimicry (VM), a malignant tumour‐specific non‐endothelial vascular network, provides oxygen and nutrients to tumour cells and facilitate tumour progression. Tao et al showed that lncRNA TP73‐AS1 was upregulated in VM positive TNBC tissues and involved in TNBC VM formation, by binding to miR‐490‐3p and activating the miR‐490‐3p/TWIST1 axis.^126^ Besides, lncRNAs are also reported to implicate in other process of TNBC cells. Such as, LINK‐A was identified to promote TNBC glycolysis reprogramming by mediating HIF1α phosphorylation at Tyr 565 and Ser 797.^127^ Tracy et al revealed that lncRNA MANCR significantly inhibited DNA damage and regulated genomic stability of TNBC.^128^

## 5 LNCRNA ACTS AS BIOMARKER FOR DIAGNOSIS AND PROGNOSIS IN TNBC

Since various lncRNAs have been found to be differentially expressed in TNBC, there is increasing evidence to show lncRNAs have diagnostic or prognostic potential for clinical TNBC patients. To study the role of lncRNAs in the diagnosis and prognosis of TNBC patients, numerous researchers analysed the lncRNAs expression levels (even epigenetic level) in TNBC versus non‐TNBC patients or healthy controls from tissues specimens, plasma (circulating lncRNA), exosome lncRNA, or micropeptide, and investigated the association with the prognosis of TNBC patients, including overall survival, disease‐free survival, lymph node metastasis, and distant metastasis. For instance, Fan et al implemented a comprehensive analysis of lncRNA expression profiles and clinical data of 1097 breast cancer samples from TCGA database. They detected 1510 differentially expressed lncRNAs in normal and TNBC samples, and 672 differentially expressed lncRNAs between non‐TNBC and TNBC samples.^129^ They identified three lncRNAs (AC091043.1, AP000924.1 and FOXCUT) maybe have strong diagnostic value for TNBC diagnosis. They also found that other three lncRNAs (AC010343.3, AL354793.1 and FGF10‐AS1) expression levels were associated with the clinical prognosis of TNBC patients.^129^ Liu et al compared the differential lncRNAs expression in the plasma of TNBC patients (n = 25), non‐TNBC patients (n = 35) and healthy controls. At last, they found that the expression levels of three lncRNAs, ANRIL, HIF1A‐AS2 and UCA1 were significantly increased in the plasma of TNBC patients, suggesting that those three lncRNAs expression may serve as TNBC‐specific diagnostic biomarkers.^130^ A recent meta‐analysis summarized the prognostic value of 24 lncRNAs from a total of 2803 TNBC patients and demonstrated that expression of nine lncRNAs (SNHG12, MALAT1, HOTAIR, HIF1A‐AS2, HULC, LINC00096, ZEB2‐AS1, LUCAT1 and LINC000173) showed a marked correlation with positive lymph node metastasis, while lncRNA MIR503HG, GAS5, TCONS_l2_00002973 showed the opposite effect.^131^ The authors also found high expression level of another seven lncRNAs (MALAT1, HIF1A‐AS2, HULC, LINC00096, ADPGK‐AS1, ZEB2‐AS1, LUCAT1) was positively correlated with distant metastasis, while patients with a high lncRNA MIR503HG expression level have lower rate of distant metastasis.^131^

DNA methylation is the best‐studied mechanism of epigenetic gene regulation.^132^ The aberrant DNA methylation statuses play an essential role in the pathological process of many cancers. It was demonstrated that TNBC tumours are genome‐wide hypomethylation compared with other subtypes and normal breast control tissues and the hypomethylation is associated with worse overall survival (OS).^133^, ^134^, ^135^ Plenty of evidence have revealed that cancer cells utilize DNA methylation as a strategy to abnormally silence a variety of genes including lncRNAs. Bermejo et al conducted an epigenome‐wide association study (EWAS) and identified that LINC00299 is high methylated in TNBC patients’ peripheral blood, making hypermethylation of LINC00299 a useful circulating biomarker for TNBC patients.^136^

LncRNAs were also be reported to predict responses to therapy, including chemotherapy, radiotherapy and immunotherapy. One study has showed that circulating lncRNA H19 was high expressed and could predict the response to neoadjuvant chemotherapy (NAC) in TNBC patients.^137^ They found patients with a pathological complete response (pCR) had lower pre‐therapeutic levels of lncRNA H19 compared with the non‐complete responders. Meanwhile, patients with higher degree of downstaging of initial tumours had lower baseline levels of lncRNA H19 among non‐complete responders.^137^ Those data suggested that circulating lncRNA H19 may be a useful marker for predicting the response to neoadjuvant chemotherapy. Another study determined that lncRNA LINK‐A could predict immunosuppression and immunotherapy resistance.^18^ TNBC patients who responded to pembrolizumab (anti‐PD‐1 immunotherapy) exhibited relatively lower expression of LINK‐A and higher CD8^+^ T‐cell infiltration compared with non‐responders. CD8^+^ T‐cell infiltration in this cohort of patients with TNBC negatively correlated with LINK‐A expression.^18^ These results implicated the potential for lncRNA LINK‐A to serve as biomarker for predicting the outcome of TNBC patients treated with immune checkpoint inhibitors. Recently, Bi et al reported that higher lncAFAP1‐AS1 expression was detected in the patients with local recurrence, using the surgically resected tumour tissues of TNBC patients receiving postoperative radiotherapy.^138^They also found higher lncAFAP1‐AS1 expression was correlated with poor disease‐free survival and overall survival of TNBC patients.^138^ These results demonstrated that high lncAFAP1‐AS1 expression is associated with radio‐resistance of TNBC patients, and the expression level of lncAFAP1‐AS1 in tumour tissues could be used to predict the outcome of TNBC radiotherapy.

**FIGURE 6:**
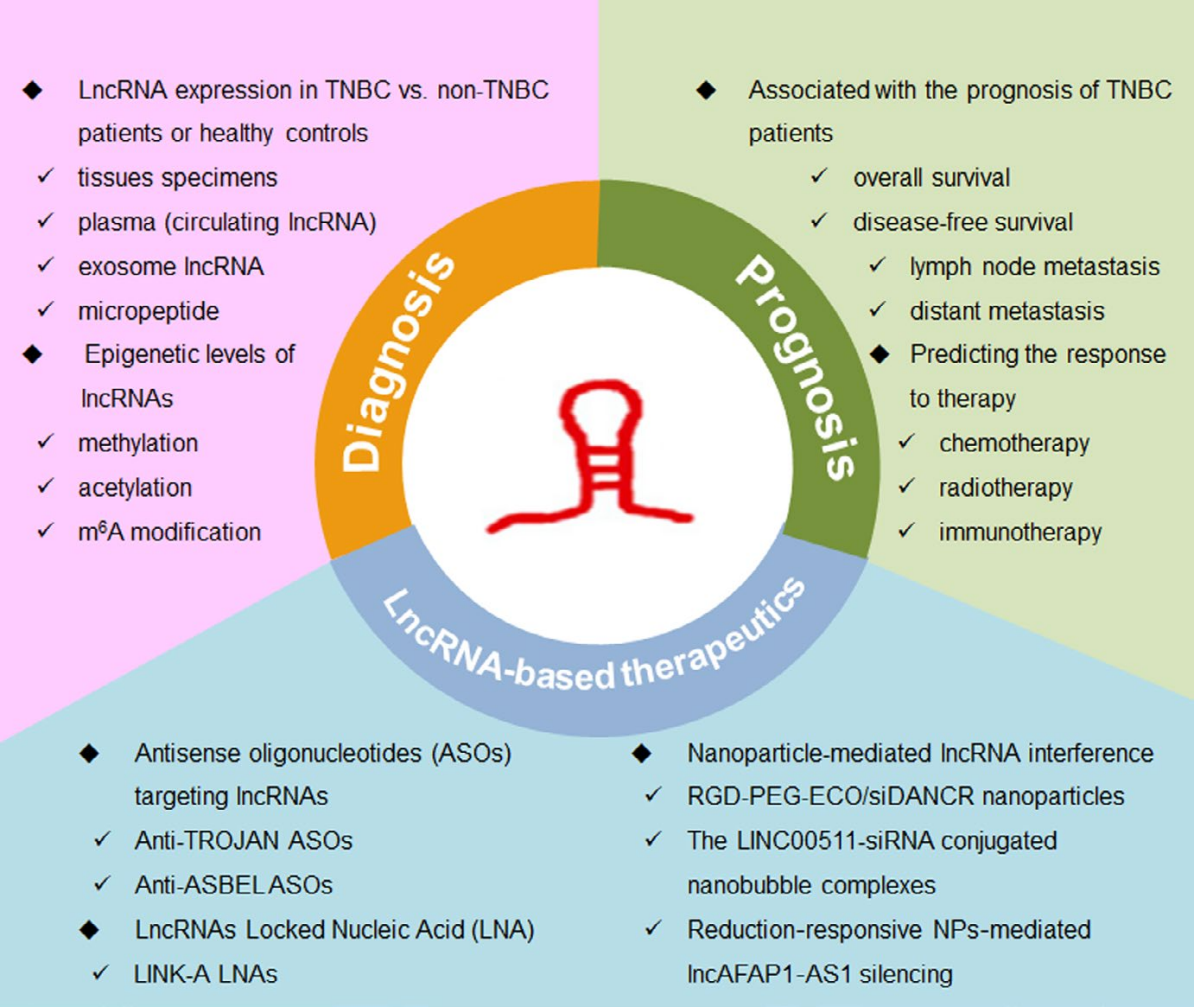
lncRNA act as biomarker for diagnosis and prognosis in TNBC

In conclude, it was noticeable that lncRNAs might be more reliable diagnostic and prognostic biomarkers for TNBC patients as a result of its aberrant expression in tumorigenesis (Figure 6). However, in the future, lncRNA diagnosis and prognosis biomarker studies will need to specify more focus on the serum circulating lncRNA and predicting the response to therapy, including chemotherapy, radiotherapy, targeted therapy and immunotherapy. Additionally, further investigation of a larger patient population is necessary to confirm the diagnostic and prognostic evaluation of lncRNAs in TNBC patients.

## 6 LNCRNA‐BASED THERAPEUTICS IN TNBC

Since lncRNAs play crucial roles in the biological processes and tumorigenesis and abnormal expression of lncRNAs occur in multiple human cancers, this presents with lncRNA‐based therapeutics possibility to correct this dysregulation. Recently, accumulating studies indicating the significance of lncRNAs in the regulation of TNBC development and drug resistance accelerated the investigation to explore the potential lncRNAs‐based therapeutics in TNBC (Figure 6).

Antisense oligonucleotides (ASOs) were able to modulate RNA processing and protein expression through different mechanisms, making them able to serve as a variety of molecular targets.^139^ Recently, advancements of ASO structure and chemical modifications greatly improved the advantage and effectiveness of ASOs to act as precious tools to understand disease mechanisms and as valuable therapeutics for disease intervention.^140^ Moreover, many ASOs are undergoing clinical trials, taking advantage of the various mechanisms and synthetic structures now available for the design of ASOs‐based therapies.^140^ ASOs also were used to inhibit the expression of lncRNAs for lncRNA‐based therapeutics in TNBC.

LncRNA TROJAN was shown to promote TNBC proliferation and metastasis, and correlated with poor patient survival.^17^ Jin et al designed eight ASOs targeting TROJAN and transfected TNBC cells with ASOs without using any transfection reagents to simulate in vivo conditions. After using anti‐TROJAN targeted therapy in an intravenous xenograft mouse model, they observed that lung metastasis nodules were significantly reduced in ASO‐treated group than the control group.^17^ Meanwhile, they also found that the ASO toxicity was limited after detecting the murine blood biochemical indexes.^17^ Taken together, these results demonstrated that modification of lncRNA TROJAN by ASO treatment maybe a novel therapeutic approach for TNBC clinical patients.

LncRNA ASBEL has been identified as an antisense transcript of BTG3 gene, which encodes an anti‐proliferation protein and is remarkable down‐regulated in TNBC. A number of single‐stranded modified ASOs were designed, synthesized and screened for specific lncRNA ASBEL knockdown. And anti‐ASBEL ASOs were reported to play a significant tumour suppressive role in TNBC by effective down‐regulating lncRNA ASBEL.^141^ Besides, Vidovic et al revealed that ASO targeting lncRNA NRAD1 could reduce tumour growth and inhibit tumour cells to acquire and maintain stem cell characteristics in TNBC.^122^

Locked Nucleic Acid (LNA) is a novel, third generation DNA analogue that has the potential to impact strongly on the future development of a diversity of nucleic acid‐based technologies.^142^ LncRNA LINK‐A has been characterized as an oncogenic lncRNA in TNBC by activating HIF1α.^127^ Recently, Hu et al reported that treatment with LINK‐A LNAs could repress cell proliferation in TNBC cells, but not non‐TNBC cells.^18^ They also found that LINK‐A LNAs‐treated MMTV‐Tg mice exhibited inhibited tumour growth and reduced lung metastasis compared with scramble LNAs‐treated mice.^18^ Besides, treatment with LINK‐A LNAs could improve the protein stability of the antigen peptide‐loading complex (PLC) components and major histocompatibility complex (MHC) class I complex, resulting in sensitization of mammary gland tumours to immunotherapy. And LINK‐A LNAs treatment could improve CD8+/CD3 + T‐cell infiltration and cytotoxicity, while tumour growth was synergistically suppressed by a combinatorial treatment of LINK‐A LNAs and immune checkpoint blockers (ICBs).^18^ Therefore, LINK‐A may act as a powerful biomarker for predicting the prognosis of TNBC patients who received immunotherapy, and targeting LINK‐A could further sensitize TNBC to immune checkpoint inhibitors.

RNA nanotechnology is a rapidly evolving field that has emerged as a novel vector system for targeted therapy in various human diseases.^143^, ^144^, ^145^ RNA nanoparticle‐based targeted therapy through inhibition of non‐coding RNA has also been reported in the treatment for human cancer.^146^ LncRNA DANCR was reported to be significantly overexpressed and promote cell proliferation, invasion, and CSC‐like phenotypic traits in TNBC.^56^, ^98^, ^120^ Vaidya et al formulated tumour‐targeting RGD‐PEG‐ECO/siDANCR nanoparticles via self‐assembly of multifunctional amino lipid ECO, cyclic RGD peptide‐PEG and siDANCR for systemic delivery.^147^ The nanoparticle‐mediated RNA interference (RNAi) of the oncogenic lncRNA DANCR demonstrated effective TNBC therapy. They found that DANCR expression was 80%‐90% knockdown after treatment with the therapeutic RGD‐PEG‐ECO/siDANCR nanoparticles in TNBC cells, indicating efficient intracellular siRNA delivery and sustained target silencing. Moreover, the RGD‐PEG‐ECO/siDANCR nanoparticles mediated significant reduction in TNBC cell proliferation, invasion, migration, survival and tumour spheroid formation, suggesting excellent in vitro therapeutic efficacy. Furthermore, the RGD‐PEG‐ECO/siDANCR nanoparticles TNBC xenografts in nude mouse model also led to suppression of TNBC progression with no overt toxic side‐effects, which demonstrated the efficacy and safety of the nanoparticle therapy. Similarly, Wu et al structured a novel theranostic agent loaded with LINC00511‐siRNA to deliver siRNA, and detected the responses of drug sensitivity in TNBC.^148^ They demonstrated that the combination of low‐frequency ultrasound (LFUS) irradiation and nanobubble complexes was regarded as an efficient and safe method for siRNA transfection.^148^ Recently, another study engineered a reduction‐responsive nanoparticle (NP) platform for effective lncAFAP1‐AS1 siRNA (siAFAP1‐AS1) delivery, and reported that systemic delivery of siAFAP1‐AS1 with the reduction‐responsive NPs can synergistically reverse radio‐resistance by scavenging intracellular glutathione, leading to a dramatically enhanced radiotherapy effect in both xenograft and metastatic TNBC tumour models.^138^ Overall, these results demonstrate that this RNA nanoparticle‐based targeted therapy by nanoparticle‐mediated modulation of onco‐lncRNAs is a promising approach that utilizes chemically modified RNPs for tumour‐specific targeting and lncRNA inhibition that will be beneficial in TNBC and other cancers setting where lncRNA knockdown is desired for a better clinical output.^138^, ^147^, ^148^

## 7 CONCLUSIONS AND FUTURE PROSPECTS

Overall, recent evidences suggest that many lncRNAs were abnormal expressed and characterized as biomarkers for diagnosis and prognosis in TNBC. LncRNAs have been identified to involve in the regulation of pathological and physiological processes of TNBC cells, including cell proliferation, apoptosis, EMT, metastasis and therapy resistance. The functional lncRNAs and their regulators hold the potential for development of novel lncRNA‐based therapeutics in clinical TNBC treatment, using ASOs, LNA or RNA nanotechnology targeting lncRNA.

In the future, (a) lncRNA diagnosis and prognosis biomarker studies will need to specify more focus on the serum circulating lncRNA and predicting the response to therapy, including chemotherapy, radiotherapy, targeted therapy and immunotherapy. (b) high throughput next generation sequencing (NGS) used for lncRNA profiling has identified a lot of differential lncRNAs in TNBC *versus* non‐TNBC tissues. However, further comprehensive functional studies of the identified TNBC‐related lncRNAs are needed. (c) Since lncRNAs play important roles in the multiple process of TNBC development, the mechanism of the regulation of abnormally expressed lncRNAs should also need to be investigated in the future, (d) almost all of the lncRNAs‐related studies in TNBC are focused on cell lines. Future studies can play more attention in the clinical TNBC patients or animal models. (e) lncRNA‐based therapeutics in TNBC have been developed by numerous researchers. However, the technology used for current lncRNA‐based therapeutics is focus on antisense oligonucleotides to inhibit the expression of onco‐lncRNAs. More technology, such as RNA nanotechnology, may hold the potential for development of novel therapeutics in clinical TNBC treatment. Furthermore, other emerging technology through lncRNA replacement therapy to restore levels of tumour‐suppressor lncRNAs could also be developed in the future.

As lncRNAs play significant roles in the TNBC tumorigenesis, further characterization of this category of molecules to uncover their potential roles as therapeutic targets, diagnosis and prognosis biomarkers for TNBC are an important priority for the clinical TNBC treatment.

## CONFLICT OF INTEREST

The authors declare that they have no conflict of interest.

## AUTHORS' CONTRIBUTIONS

WZ, XG and JT conceived the study and wrote the manuscript.

## Data Availability

The data that support the findings of this study are available from the corresponding author upon reasonable request.
